# Intranasal Vaccination with Leishmanial Antigens Protects Golden Hamsters (*Mesocricetus auratus*) Against *Leishmania (Viannia) braziliensis* Infection

**DOI:** 10.1371/journal.pntd.0003439

**Published:** 2015-01-08

**Authors:** Luzinei da Silva-Couto, Raquel Peralva Ribeiro-Romão, Andrea Franco Saavedra, Beatriz Lilian da Silva Costa Souza, Otacílio Cruz Moreira, Adriano Gomes-Silva, Bartira Rossi-Bergmann, Alda Maria Da-Cruz, Eduardo Fonseca Pinto

**Affiliations:** 1 Laboratório Interdisciplinar de Pesquisas Médicas - Instituto Oswaldo Cruz - FIOCRUZ, Rio de Janeiro, Brazil; 2 Laboratório de Imunofarmacologia - Instituto de Biofísica Carlos Chagas Filho, UFRJ, Rio de Janeiro, Brazil; 3 Laboratório de Biologia Molecular e Doenças Endêmicas - Instituto Oswaldo Cruz - FIOCRUZ, Rio de Janeiro, Brazil; The George Washington University Medical Center, United States of America

## Abstract

**Background:**

Previous results have shown that oral and intranasal administration of particulate *Leishmania (Leishmania) amazonensis* antigens (LaAg) partially protects mice against *L. amazonensis* infection. However, vaccination studies on species of the subgenus *Viannia*, the main causative agent of cutaneous and mucosal leishmaniasis in the Americas, have been hampered by the lack of easy-to-handle bio-models that accurately mimic the human disease. Recently, we demonstrated that the golden hamster is an appropriate model for studying the immunopathogenesis of cutaneous leishmaniasis caused by *L. (Viannia) braziliensis.* Using the golden hamster model, our current study investigated whether the protective effect of intranasal immunisation with LaAg can be extended to *L. braziliensis* infection.

**Methodology/Principal Findings:**

Golden hamsters vaccinated with either two intranasal (IN) doses of LaAg (10 µg) or two intramuscular doses of LaAg (20 µg) were challenged 2 weeks post-vaccination with *L. braziliensis*. The results showed that IN immunisation with LaAg significantly reduced lesion growth and parasitic load as well as serum IgG and IgG2 levels. At the experimental endpoint on day 114 post-infection, IN-immunised hamsters that were considered protected expressed IFN-γ and IL10 mRNA levels that returned to uninfected skin levels. In contrast to the nasal route, intramuscular (IM) immunisation failed to provide protection.

**Conclusions/Significance:**

These results demonstrate for the first time that the nasal route of immunisation can induce cross protection against *L. braziliensis* infection.

## Author Summary

Leishmaniasis is a disease that is common in most tropical countries. In Brazil, the cutaneous form of the disease is highly prevalent, with approximately 28,000 new cases reported annually. *L. (Viannia) braziliensis* is the main causative agent of cutaneous leishmaniasis; however, vaccine studies against protozoans of the subgenus *Viannia* have been largely neglected, mainly due to the high resistance of most mouse strains to the infection. Here, the authors used the golden hamster, which is highly susceptible to dermotropic *Leishmania spp* infection. It was previously shown that oral and intranasal vaccination with whole *L. (Leishmania) amazonensis* antigens (LaAg) protected mice against *L. amazonensis* infection. In the present study, the authors investigated whether the protective effect of intranasal immunisation with LaAg can be extended to *L. braziliensis* infection using the golden hamster model. The results showed that intranasal immunisation with LaAg significantly reduced lesion growth and parasitic load as well as IgG and IgG2 serum levels. At the endpoint of the experiment, intranasally immunised hamsters that were considered protected expressed IFN-γ and IL10 mRNA at levels similar to those in uninfected skin. These data show that the use of a proper animal model and/or different vaccination strategies may facilitate the development of an effective vaccine against *L. braziliensis.*

## Introduction

Leishmaniasis is a neglected disease caused by intracellular protozoan parasites of the genus *Leishmania spp*, with a wide spectrum of clinical manifestations ranging from chronic cutaneous ulcers to fatal visceral disease. *L.* (*Viannia*) *braziliensis* is the most prevalent species associated with American tegumentary leishmaniasis (ATL), which constitutes a serious public health problem affecting 28,000 people annually in Brazil [1]. Despite the advent of new anti-leishmanial compounds [2], multiple injections of pentavalent antimonials, which invariably produce serious toxic side effects, still remain the first-line therapy for all forms of the disease. The problem is further aggravated by therapeutic failure along with the emergence of antimonial resistance [3].

The development of a vaccine against leishmaniasis is a long-term goal in both human and veterinary medicine. Intramuscular or intradermal injections with killed *L. (Leishmania) major* promastigotes in the absence of adjuvants in mice [4] [5] and with killed *L. amazonensis* promastigotes in mice [6] [7] and monkeys [8] actually exacerbated subsequent infections, suggesting that such formulations contain disease-inducing antigens.

Mucosal administration of disease-promoting antigens has been used as a feasible strategy to induce immunotolerance and protection against autoimmune and allergic diseases [9]. Peripheral tolerance resulting from intestinal or nasal antigen uptake formed the basis of the present work using disease-promoting parasitic antigen. However, systemic immunity may also be achieved with antigens administered through the mucosa [10]. This balance between tolerance and immunity is determined by the nature of the antigen, antigen dosag, antigen form(i.e., soluble or particulate), the route of antigen administration and the presence of adjuvants [11].

We previously demonstrated that oral vaccination with whole *L. amazonensis* antigens (LaAg) confers different strains of mice with partial protection against cutaneous leishmaniasis (CL) caused either by *L. amazonensis*, or *L. major* [6]. Compared to the oral route, the intranasal administration of LaAg protected BALB/c mice against *L. amazonensis* infection more effectively, provided convenience in its ease of administration, and required lower antigen doses [12].

Several mouse models exist for modelling immunity against *Leishmania spp*. However, vaccine studies on protozoans of the subgenus *Viannia*, the main causative agent of cutaneous and the dreadful mucosal leishmaniasis in the Americas, are hampered by the lack of viable bio-models that accurately reflect the human disease. In these mouse models, most inbred mouse strains (including C57BL/6 and BALB/c) are resistant to *L. braziliensis* infection, developing non-ulcerative lesions that spontaneously heal within 10 weeks [13], [14]. Vaccine studies attempting to induce immunological protection against *L. (Viannia)* parasites in mice using a variety of parasite antigens [15]–[17] have been conducted with limited success. Salay et al. [15] tested four different highly conserved leishmanial antigens (DNA and recombinant proteins) along with adjuvants and found that protective immunity previously afforded against experimental CL caused by *L. major* could not be reproduced against an *L. braziliensis* challenge. Therefore, the development of an effective vaccine against *L. braziliensis* infection necessitates a suitable animal model and/or different vaccination strategies.

The golden hamster (*Mesocricetus auratus*) is highly susceptible to dermotropic *Leishmania spp* infection and has been largely used as a model for visceral leishmaniasis [18]–[20]. Recently, we demonstrated that the golden hamster is also an appropriate model for studies on CL immunopathogenesis caused by *L. (V.) braziliensis* because it develops chronic skin lesions that clinically and histopathologically mirror those of humans. These features justify the use of the golden hamster model in clinical, vaccination and chemotherapy experimental protocols [21], [22].

Thus, in the present study, we proposed to evaluate the effectiveness of intranasal LaAg vaccination against *L. braziliensis* infection using the golden hamster as a model.

## Materials and Methods

### Ethics statement

This study was carried out in strict accordance with the recommendations in the Guide for the Care and Use of Laboratory Animals of the Brazilian National Council of Animal Experimentation (http://www.cobea.org.br). The protocol was approved by the Committee on the Ethics of Animal Experiments of the Institutional Animal Care and Use Committee at the Fundação Oswaldo Cruz (FIOCRUZ) (CEUA protocol LW 11/11).

### Animals

Seventy-two outbred adult female (6–8 weeks old, weighing 80–90 grams) golden hamsters (*M. auratus*) from the animal facilities at FIOCRUZ, were used. Four independent experiments were performed.

### Parasites

*L. amazonensis* promastigotes (MHOM/BR/75/Josefa) in early stationary growth phase were used for vaccine preparation. *L. braziliensis* promastigotes (MCAN/BR/98/R619) in stationary growth phase were cultured in supplemented Schneider's *Drosophila* medium (Sigma Chemical Co., St. Louis, USA) and used until the third *in vitro* passages for infection. Disrupted antigens of *L. braziliensis* (MHOM/BR/75/2903) promastigotes (Lb-Ag) were obtained for immunological studies.

### Vaccine – *L. amazonensis* antigen (LaAg)

LaAg was prepared as previously described (6). Briefly, *L. amazonensis* promastigotes were washed three times by centrifugation, resuspended at 2×10^8^ parasites/mL in phosphate-buffered saline (PBS) and submitted to 15 cycles of freezing and thawing. The resulting cell lysate was termed LaAg. One millilitre of LaAg contained 970 µg of protein, as measured by the Lowry assay [23]. Sample aliquots were stored at −20° until use.

### Immunisation

Hamsters held upwards either received nasal instillations of 10 µg of LaAg (IN LaAg) in 20 µL of PBS (10 µL in each nostril) (n27 animals) using a fine tip attached to a micropipette, or receive two intramuscular injections of 20 µg of LaAg (IM LaAg) in 100 µL of PBS (n = 18 animals) into the thighs. Animals were boosted fourteen days later using the same vaccine dosage. Controls received PBS (n = 27 animals).

### Infection

Two weeks after the second vaccinating dose, the hamsters were infected intradermally with 1×10^5^ *L. braziliensis* promastigotes (section 2.2) in the dorsal hind paw. Lesion sizes were measured every 7 days with a dial caliper (Mitutoyo, America Corporation, São Paulo, Brazil) and expressed as the difference between the thickness of the infected and uninfected paws.

### Parasite loads

At the experimental endpoint,the animals were euthanised, and fragments of the infected paw were cut off, weighed and individually homogenised in 1 mL of PBS using a tissue grinder. Parasite load was then analysed by the limiting dilution assay [24].

### Quantification of anti-*Leishmania spp* antibodies

Anti-leishmanial IgG and IgG2 levels in plasma samples were determined by the ELISA assay, as described elsewhere (21). Plasma samples were diluted 1∶5000 for IgG and 1∶200 for IgG2 and detected by horseradish peroxidase-labelled goat anti-hamster IgG (Santa Cruz Biotechnology, Santa Cruz, California, USA) and biotin–conjugated mouse anti-Syrian hamster IgG2 (Becton Dickinson, New Jersey, USA).

### Tissue cytokine mRNA expression by real time RT PCR

Skin of infecte paws were collected in RNAlater (Ambion, Life Technologies, Carlsbad, California, USA) and frozen at −20°C until use. Total RNA was extracted from 20–30 mg of tissue using an RNeasy kit (Qiagen, Austin, Texas, USA). RNA samples were treated with RQ1 RNase-free DNase (Promega Corporation, Madison WI, USA) and reverse transcribed using a High Capacity Reverse Transcription kit (Applied Biosystems, Foster City, CA, USA). For real-time qPCR (RT-qPCR) assays of target genes, duplicate wells each containing 2 µL of cDNA and 7.5 µL of TaqMan Universal PCR Mastermix were used in a final reaction volume of 15 µL and analysed with ABI Prism 7500 Fast Real-Time PCR equipment (Applied Biosystems, USA). The sequences and concentrations of primers and probes for the hamster target genes (*IFNG* and *IL10*) as well as for the RT-qPCR cycling conditions used in this study have been previously described [25] Gene expression (Fold Change) was calculated by relative quantification using the comparative Ct method (ΔΔCt), as described by Livak & Schmittgen [26], using the golden hamster GAPDH and γ-actin genes as housekeeping controls. Subsequently, gene expression in infected animals was normalised to uninfected control, and the final results are expressed as 2^-ΔΔCt^ (fold change).

### Statistical analysis

The data were analysed by the Mann-Whitney test and Spearman's rank-correlation using GraphPad Prism software version 5.00 for Windows (GraphPad Software, San Diego, CA, USA). The results are expressed as the mean ± standard error of the mean and median. p<0.05 was considered statistically significan.

## Results

### Intranasal, but not intramuscular, LaAg vaccination confers protection against *Leishmania (Viannia) braziliensis* infection

**Figure 1:**
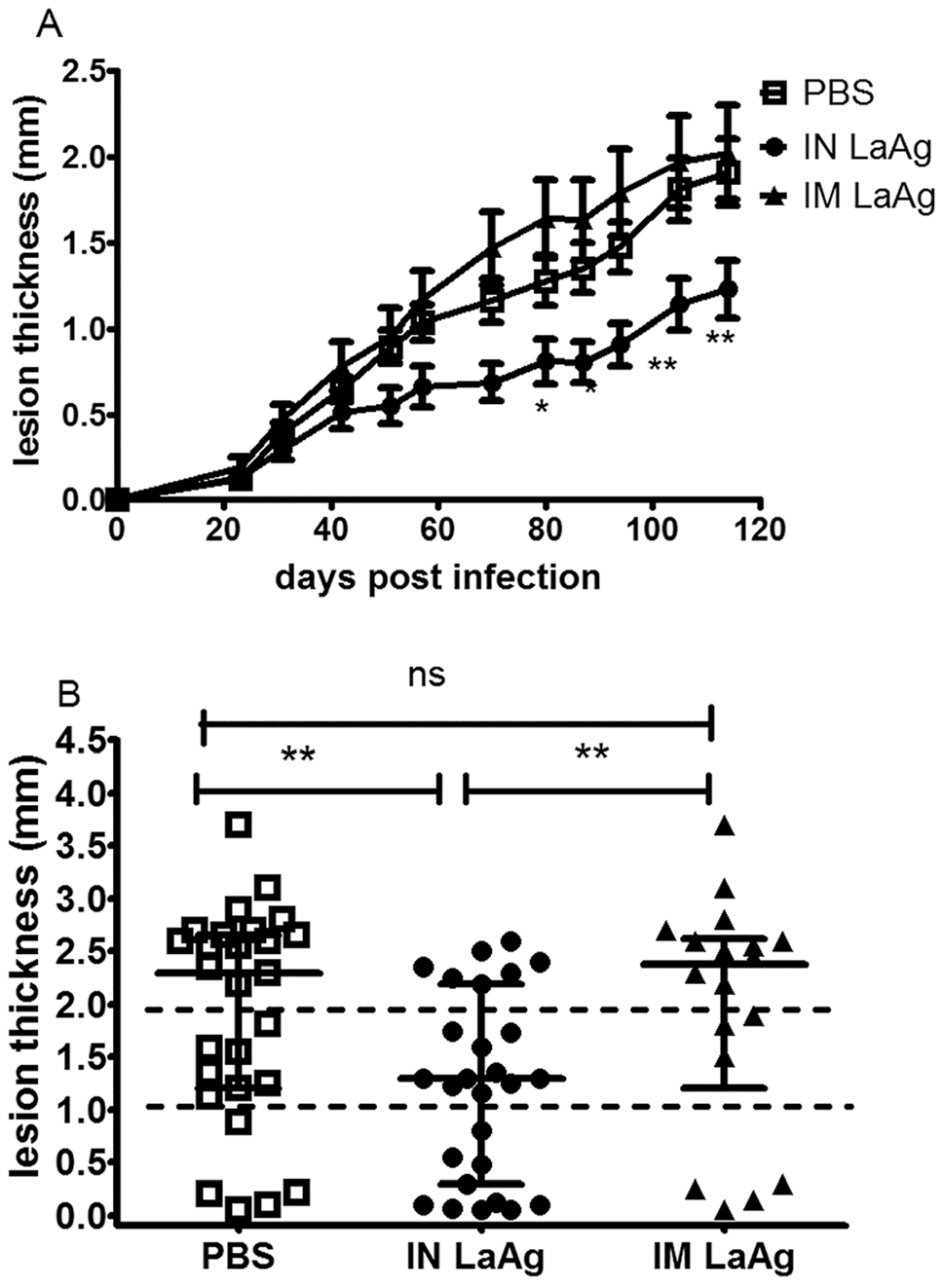
Effectiveness of intranasal (IN) and intramuscular (IM) *L. amazonensis* antigens (LaAg) vaccination against subsequent infection with *L. braziliensis.* Golden hamsters (6–8 per group) were vaccinated with either 10 µg of LaAg intranasally (•) or 20 µg of LaAg intramuscularly (▴) followed by a booster vaccination 14 days later. Controls received PBS (□). Two weeks after the booster vaccination, animals were infected in the paw with 1×10^5^ *L. braziliensis*. (A) Lesion thickness was scored on the indicated days. Means ± standard errors of the means (SEM), * p≤0.05, ** p≤0.01. (B) On day 114 post-infection, the lesion thickness of individual feet was shown as individual values, with the horizontal bars representing the median values. Data are from four independent experiments.

The capacity of IN LaAg to protect hamsters against *L. braziliensis* was first compared with the classical intramuscular route. At day 50 after parasite challenge, IN LaAgimmunised hamsters already presented reduced paw thickness than that of IM-immunised hamsters or infected controls. From day 84 until the experimental endpoint on day 114, the difference was statistically significant (p<0.05) (Fig.1A). At this time, the percentage of animals presenting no lesions or nodular lesions less than 1 mm thick was 37.0% (10 out of 27 animals) in the IN LaAg group, compared to 22% (LaAg IM = 4 out of 18 animals) and 19% (PBS control  = 5 out of 27 animals) in the other two groups (Fig. 1B). Moreover, only 26% (7 out of 27) of animals from the IN LaAg group presented ulcerated lesions greater than 2 mm thick, compared to 61% (LaAg IM = 11 out of 18 animals) and 55% (PBS control  = 15 out of 27 animals) in the other two groups (Fig. 1B). The development of lesions was similar between the IM LaAg and PBS control groups (Fig. 1A).

### Intranasal vaccination with LaAg decreases parasite loads

**Figure 2:**
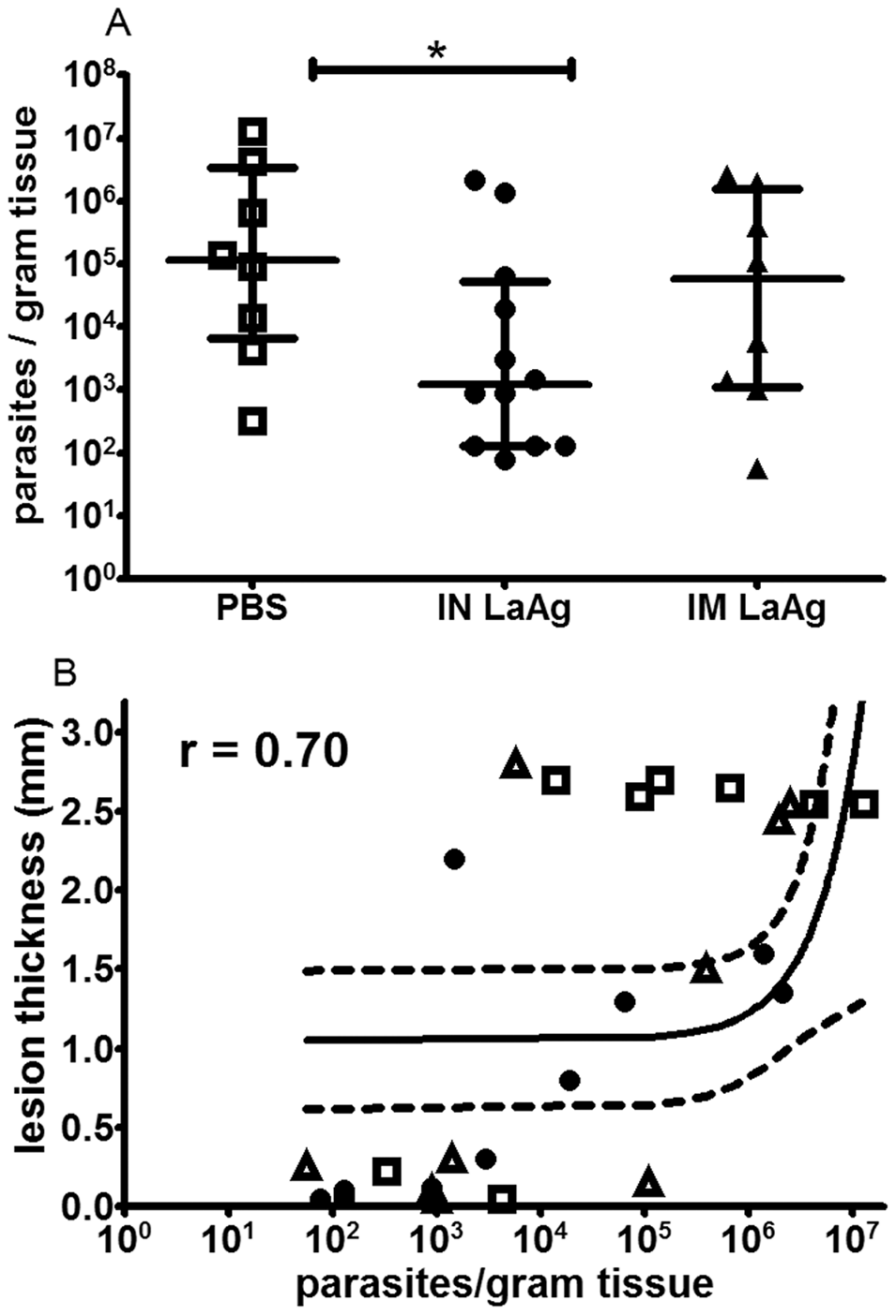
Parasite loads from skin lesions of hamsters immunised with crude *L. amazonensis* antigens (LaAg) and infected with *L. braziliensis*. Golden hamsters (4–6 per group) were immunised with IN LaAg (•), IM LaAg (▴), or received PBS (□). Two weeks after the last immunisation, hamsters were challenged with *L. braziliensis*. A) Paw skin parasite loads were determined 114 days post-infection via a limiting dilution assay. B) Correlation between skin parasite load and lesion thickness (r = 0.70, p<0.0001). Each point represents one animal. The horizontal bars represent the median values, * p≤0.05. Data are from two independent experiments.

To assess whether the decreased lesion growth observed in Fig. 1 was a result of decreased parasite growth, the number of parasites in the lesions was quantitated at the end of the experiment (day 114). The IN-immunised animals that were considered protected (no lesion or nodular lesion with thickness <1 mm) also presented lower parasite loads compared to the PBS control group (p<0.05). However, no significant difference was observed between PBS controls and IM LaAg animals (p>0.05) (Fig. 2A). Plotting readings from individual animals, a positive correlation was observed between paw lesion thickness and parasite load when expressed as the number of parasites/gram of infected paw (r = 0.70, p<0.0001) (Fig. 2B). This finding indicates that in contrast to IM LaAg vaccination that did not alter the parasitological parameters, IN LaAg vaccination led not only to smaller lesion growth but also to lower parasite burden.

### Intranasal LaAg vaccination leads to decreased anti-leishmanial IgG and IgG2 levels

**Figure 3:**
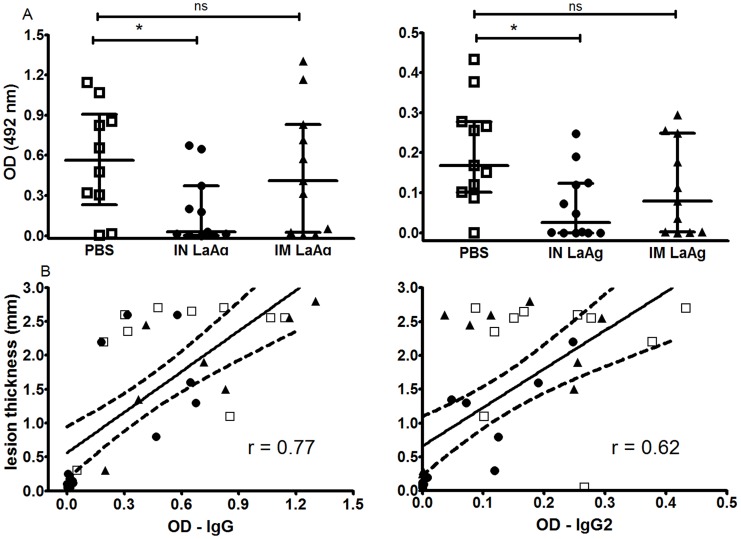
Anti-*Leishmania* IgG and IgG2 serum levels in hamsters immunised with *L. amazonensis* antigens (LaAg) and infected with *L. braziliensis*. Golden hamsters (5–6 per group) were immunised with IN LaAg (•), IM LaAg (▴) or PBS (□). Two weeks after the last immunisation, hamsters were challenged with *L. braziliensis*. A) IgG and IgG2 serum levels were determined 114 days post-infection by ELISA. B) Correlation between lesion thickness and anti-leishmanial IgG (r = 0.78, p<0.0001) or IgG2 (r = 0.62, p<0.0001) serum levels. Each point represents one animal. The horizontal bars represent the median values, * p≤0.05. Data are from two independent experiments.

Parasite-specific antibodies are associated with progressive cutaneous and visceral leishmaniasis in hamster models [21], [27], [28]. The animals immunised with IN LaAg that were considered protected also presented lower levels of anti-*Leishmania* IgG and IgG2 compared to those of the control group (Fig. 3A). However, no significant difference was observed between the PBS control and IM LaAg groups. A positive correlation was observed between paw lesion size and anti-leishmanial IgG (r = 0.78, p<0.0001) or IgG2 (r = 0.62, p<0.0001), indicating that IgG and IgG2 levels are related to the severity of the infection(Fig. 3B) and are not induced when animals are IN vaccinated with LaAg.

### Intranasal vaccination with LaAg decreases IFN-γ in *Leishmania braziliensis*-infected skin

Due to its ability to activate macrophages to kill parasites, IFN-γ is a key cytokine that controls *Leishmania spp* infection. However, high levels of this cytokine are also implicated in tissue damage in humans [29], [30]. However, IL10 is involved in mediating parasite persistence, regulating Th2 cell expansion and controlling cell-mediated lesion development in leishmaniasis [30].

Herein, the transcripts coding IFN-γ and IL10 were quantitated in the skin lesions of the infected hamsters by RT-qPCR, and the results are expressed as fold increase relative to uninfected paws.

**Figure 4:**
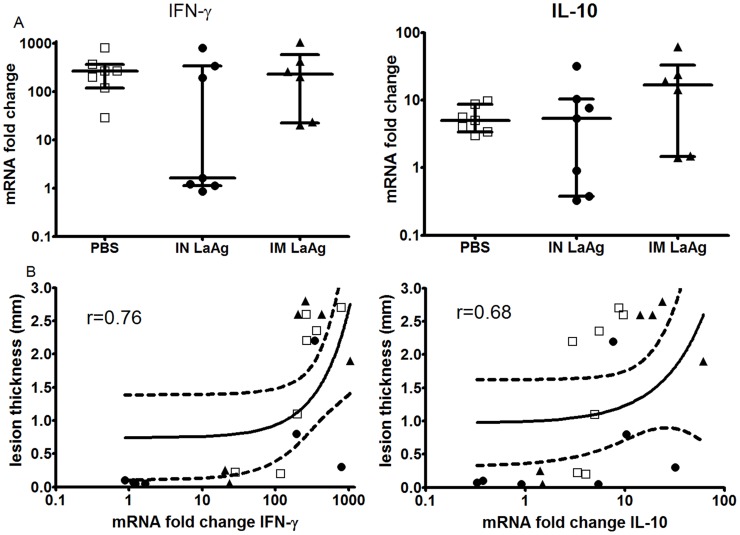
Cytokine mRNA expression in skin of hamsters immunised with *L. amazonensis* antigens (LaAg) and infected with *L. braziliensis*. Golden hamsters (6–7 per group) were immunised with IN LaAg (•), IM LaAg (▴) or PBS (□). Two weeks after the last immunisation, hamsters were challenged with *L. braziliensis*. (A) mRNA gene expression for IFN-γ and IL-10 in skin lesions was determined 114 days post-infection. (B) Correlation between lesion thickness and IFN-γ (r = 0.76, p<0.0001) or IL-10 (r = 0.68, p<0.0001). Each point represents one animal. The horizontal bars represent the median values. Symbols represent the fold change in cytokine expression relative to that of uninfected hamsters. Data shown are from one of two independent experiments.

The IN LaAg-immunised group presented a dual profile of *IFNG* expression: the four animals having the smallest lesions presented gene expression similar to that of uninfected skin (fold increase close to 1), while the three animals that had larger lesions were those with the highest IFN-γ gene expression levels in their skin lesion comparable to that of the IM LaAg and non-immunised PBS groups (Fig. 4A). A positive correlation was observed between paw lesion size and IFN-γ in skin lesions (r = 0.76, p<0.0001) (Fig. 4B). *IL10* expression was similar between all three groups (Fig. 4A). Intriguingly, three animals from the IN LaAg group presenting smaller lesions also showed low *IL10* expression (Fig. 4B). These findings showed that on day 114 post-infection, there was no correlation between protection induced by IN LaAg and IFN-γ and IL-10 gene expression.

## Discussion

The majority of studies aimed at developing an effective *Leishmania spp* vaccine have been conducted for experimental CL caused mainly by *L. major* or *L. amazonensis*. *L. braziliensis*, which is the main causative agent of cutaneous and mucosal leishmaniasis in the Americas, has been largely neglected in the context of vaccine development mainly due to the unsuitability of the mouse model. Moreover, candidate antigens - such as the leishmanial homologue of receptors for activated C kinase protein (LACK), thiol-specific antioxidant (TSA), *Leishmania* spp elongation and initiation factor (LeIF) and *L. major* stress-inducible protein 1 (LmST1) - all of which induced protection against *L. major*, failed to prevent *L. braziliensis* infection in mice [15].

In this work, the results demonstrated for the first time, that the intranasal administration of adjuvant-free crude *L. amazonensis* antigens is efficacious against *L. braziliensis* infection in the golden hamster model. Comparing the IN and IM routes of immunisation, it was also observed that protective immunity induced by LaAg is critically dependent upon the route of immunisation, as previously observed in the murine model of infection with *L. amazonensis* [6], [7], [12].

No mucosal adjuvant was used with LaAg because there is currently none approved for human use [31]. The presence of particulate antigens in the LaAg may possibly have functioned as intrinsic adjuvants.

Although protective immunity can be acquired following natural infection, no vaccine against leishmaniasis has been licensed for human use. One of the most extensively studied human vaccines is Leishvacin comprised of whole-killed promastigotes of *L. amazonensis*, which has passed Phase 1 and Phase 2 clinical trials for safety and immunogenicity in human volunteers upon systemic administration [32]. Despite this, its efficacy was not confirmed after a controlled phase III clinical trial in Colombia [33]. Recently, clinical trials of the first defined vaccine for leishmaniasis show that the LEISH-F1 + MPL-SE vaccine, a multisubunit recombinant *Leishmania* vaccine composed of the recombinant Leishmania polyprotein LEISH-F1 (formerly known as Leish-111f) antigen and the MPL-SE adjuvant, is safe and immunogenic in healthy subjects [34], [35].

Considering that hamsters present an outbred genetic background, it is expected that individual characteristics have an important role in influencing different clinical outcomes of the disease, thereby mimickingclinical outcomes and immune response observed in human disease.

Due to the lack of a broad range of hamster-specific immunological reagents in the market [36], this work focused on the study of the immune response at the transcriptional level using real time RT PCR and on the association between antibody levels and disease protection.

We observed an association between IgG levels and disease severity, similar to the results previously shown in *L. panamensis* infection [27]. Moreover, we observed that the animals immunised with IN LaAg that were considered protected also presented lower levels of anti-*Leishmania* IgG and IgG2.

Although the presence of inflammatory cytokines (such as IFN-γ and TNF) is critical for the control of parasite dissemination, an exaggerated Th1 response has been associated with the severe tissue inflammation observed in CL lesions [29], [37], [38].

Herein, it was observed that at 114 days post-infection, the animals that had larger lesions were those with the highest IFN-γ levels in their skin lesions. Our results suggest that in this experimental model, IFN-γ gene expression may mediate immunopathology and that the high IFN-γ levels in later stages of infection may be an attempt to control the elevated parasitic load in the skin lesion.Although post-transcriptional or post-translational modifications may additionally regulat the target genes of interest, different publications have described a good correlation between cytokine gene expression quantified by RT-qPCR and cytokine secretion as measured by ELISA [39].

As noted, the animals from the IN LaAg group that presented smaller lesions also showed a lower parasite load that was associated with lower IFN-γ mRNA expression and lower IgG2 levels. These results suggest that at the endpoint of infection, the activation of macrophages to kill parasites may involve cytokines other than IFN-γ.

The presence of parasites in the skin the animals considered protected can be the ideal situation in order to maintain CD8 T cell memory. Recent reports indicate a correlation between the persistence of parasites at the primary infection site and the development of long-lasting immunity to reinfection [40].

It has been hypothesized that a modulation in the immune response induced by IN LaAg occurs in an early phase of the infection, which could dictate the establishment and magnitude of the chronic phase of the disease. The mechanisms underlying the protection conferred by IN LaAg could not be clearly established here, and further work is required to determine both the immunogenicity of LaAg and the immune response in the skin during the early phase of experimental *L. braziliensis* infection.

In the present study, we described for the first time that nasal immunisation with whole/crude *L. amazonensis* antigens (LaAg) can protect golden hamsters against *L. braziliensis* infection. That such a vaccination strategy effectively induced cross-protection against another *Leishmania* species suggests that it may have a broad spectrum of application.
